# Contactless Vital Signs Measurement System Using RGB-Thermal Image Sensors and Its Clinical Screening Test on Patients with Seasonal Influenza

**DOI:** 10.3390/s20082171

**Published:** 2020-04-13

**Authors:** Toshiaki Negishi, Shigeto Abe, Takemi Matsui, He Liu, Masaki Kurosawa, Tetsuo Kirimoto, Guanghao Sun

**Affiliations:** 1 Graduate School of Informatics and Engineering, The University of Electro-Communications, 1-5-1 Chofugaoka, Chofu, Tokyo 182-8585, Japan; negishi@secure.ee.uec.ac.jp (T.N.); kurosawa@uec.ac.jp (M.K.); kirimoto@ee.uec.ac.jp (T.K.); 2 Takasaka Clinic, Fukushima 973-8407, Japan; rsh71841@nifty.com; 3 Graduate School of System Design, Tokyo Metropolitan University, Tokyo 191-0065, Japan; tmatsui@tmu.ac.jp; 4 School of Materials Science and Engineering, Harbin University of Science and Technology, Harbin 150000, China; he.liu@hrbust.edu.cn

**Keywords:** contactless measurement, vital signs, RGB-thermal image processing, infection diseases

## Abstract

*Background:* In the last two decades, infrared thermography (IRT) has been applied in quarantine stations for the screening of patients with suspected infectious disease. However, the fever-based screening procedure employing IRT suffers from low sensitivity, because monitoring body temperature alone is insufficient for detecting infected patients. To overcome the drawbacks of fever-based screening, this study aims to develop and evaluate a multiple vital sign (i.e., body temperature, heart rate and respiration rate) measurement system using RGB-thermal image sensors. *Methods:* The RGB camera measures blood volume pulse (BVP) through variations in the light absorption from human facial areas. IRT is used to estimate the respiration rate by measuring the change in temperature near the nostrils or mouth accompanying respiration. To enable a stable and reliable system, the following image and signal processing methods were proposed and implemented: (1) an RGB-thermal image fusion approach to achieve highly reliable facial region-of-interest tracking, (2) a heart rate estimation method including a tapered window for reducing noise caused by the face tracker, reconstruction of a BVP signal with three RGB channels to optimize a linear function, thereby improving the signal-to-noise ratio and multiple signal classification (MUSIC) algorithm for estimating the pseudo-spectrum from limited time-domain BVP signals within 15 s and (3) a respiration rate estimation method implementing nasal or oral breathing signal selection based on signal quality index for stable measurement and MUSIC algorithm for rapid measurement. We tested the system on 22 healthy subjects and 28 patients with seasonal influenza, using the support vector machine (SVM) classification method. *Results*: The body temperature, heart rate and respiration rate measured in a non-contact manner were highly similarity to those measured via contact-type reference devices (i.e., thermometer, ECG and respiration belt), with Pearson correlation coefficients of 0.71, 0.87 and 0.87, respectively. Moreover, the optimized SVM model with three vital signs yielded sensitivity and specificity values of 85.7% and 90.1%, respectively. *Conclusion*: For contactless vital sign measurement, the system achieved a performance similar to that of the reference devices. The multiple vital sign-based screening achieved higher sensitivity than fever-based screening. Thus, this system represents a promising alternative for further quarantine procedures to prevent the spread of infectious diseases.

## 1. Introduction

Emerging infectious diseases are serious threats to global health. During the last two decades, there have been travel-related outbreaks of infectious diseases, such as severe acute respiratory syndrome and novel Coronavirus (2019-nCoV), around the world in 2003 and 2019 [1,2]. To contain the outbreak of emerging viral diseases, infrared thermography (IRT) has been applied for fever screening of passengers with suspected infection in many international quarantine stations [3,4,5]. IRT is an effective method for measuring elevated body temperature. However, monitoring body temperature alone is insufficient for accurate detection of infected patients, as IRT monitoring facial surface temperature can be affected by many factors such as antipyretic consumption [6]. The positive predictive values of fever-based screening using IRT vary from 3.5% to 65.4%, indicating the limited efficacy for detecting symptomatic passengers [7].

**Figure 1:**
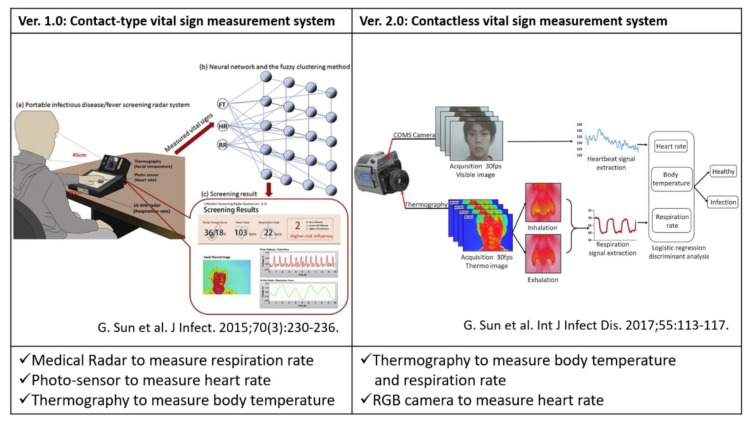
Contact and contactless vital sign measurement systems for infection screening. The figures were with copyright permission [8,10].

To overcome the drawbacks of fever-based screening, we previously proposed a screening method based on simultaneously measuring three vital signs—body temperature, heart rate (HR) and respiration rate (RR)—using multiple sensors, that is, medical radar, thermograph, photo-sensor and RGB cameras [8,9,10]. These three vital signs were included in the criteria of the systemic inflammatory response syndrome [11]. Symptoms of the most infectious diseases tend to include an elevated HR and RR; hence, a screening that combines these three vital signs will improve the precision of detecting patients with such symptoms. Therefore, we developed contact and contactless vital sign measurement systems to investigate the feasibility of our screening method (Figure 1). In brief, the contact-type system (Ver.1.0) comprises three sensors, that is, medical radar, photo-sensor and thermograph [8]. The medical radar detects tiny body surface movements caused by respiration, the thermograph measures the highest temperature of the face and the photo-sensor monitors pulse waves to calculate the HR. To enable a completely contactless system (Ver.2.0), we combined RGB and the thermal image to extract multiple vital signs from the facial image [10]. The RR can be measured by monitoring the temperature changes around the nasal and oral areas accompanying inspiration and expiration. The RGB camera measures the blood volume pulse (BVP) through variations in the light absorption from the human facial area. We tested the systems on patients with seasonal influenza and dengue fever and the results indicate a sensitivity ranging from 81.5–98% [12].

In this study, to promote the widespread use of our vital sign-based infection screening method, we enhanced the function of the Ver.2.0 contactless system to enable a stable, reliable and real-time system. We improved the stability of HR and RR measurement with the RGB-thermal image fusion approach for a highly reliable facial region-of-interest (ROI) tracking [13]. Moreover, we focused on improving the robustness of extracting BVP and respiration signal from the RGB camera and IRT. We proposed a signal processing method for reconstructing the BVP waveform using all RGB channels and selecting nasal or oral breathing based on signal quality index (SQI), for improving the signal-to-noise ratio. To enable a real-time system, we implemented a multiple signal classification (MUSIC) algorithm to estimate the pseudo-spectrum from limited time-domain BVP and respiration signals within 15 s [14]. Finally, we tested the system on 22 healthy subjects and 41 patients with influenza-like symptoms (28 diagnosed influenza patients and 13 undiagnosed patients).

The remainder of this paper is organized as follows. In the Section “Materials and Methods,” we describe an overview of our system and proposed signal and image processing methods. The Section “Results” contains the results of comparison between our contactless system with contact-type reference devices and screening performance on detecting influenza patients using a support vector machine (SVM). In the Section “Discussion and Conclusion,” we discuss our findings and draw conclusions.

## 2. Materials and Methods

### 2.1. Related Work on Vision based Clinical Screening

Vision-based clinical screening using RGB and thermal image sensors have recently attracted increasing attention in academia and industry. Ming-Zher Poh et al. developed a robust method for measuring HR and HRV from digital RGB video recording of skin color changes [15]. He Liu et al. proposed a novel method using dual cameras to estimate arterial oxygen saturation [16]. Philips Research has been launching an app called “*Vital Signs Camera*” in 2012. Moreover, the thermal camera-based approaches have been widely applied in clinical screening and research, such as fever screening and human pose estimation [5]. To enable such specific applications, image processing method for keypoint detection has been proposed using a stacked hourglass network and feature boosting networks [17,18,19].

### 2.2. Overview of Infectious Screening System using RGB-thermal Image Sensors

**Figure 2:**
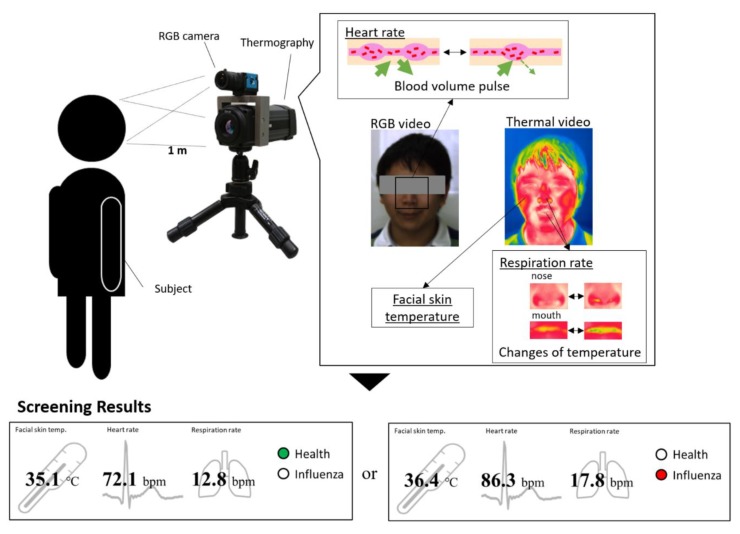
Overview of measurement principle that remotely senses multiple vital signs and an example of screening result.

In our previous work, a dual image sensor-based infectious screening system was developed for predicting the possibility of infection [10]. It comprises an RGB camera and an IRT for measuring HR, RR and body temperature. We used DFK23U618 (The Imaging Source Co. Ltd., Germany) as the RGB camera and FLIR A315 (FLIR Systems, Inc., USA) as the IRT. The visible video was recorded at a speed of 15 frames per second (fps) with a pixel resolution of 640 × 480 and the thermal video was recorded at a speed of 15 fps with a pixel resolution of 320 × 240. An RGB camera senses fluctuations in hemoglobin absorption derived from the volumetric change in facial blood vessels and obtains heartbeat signals. An IRT detects temperature changes between inhalation and exhalation in the nasal or oral area. In addition, the facial skin temperature is measured by the IRT. Multiple vital signs distinguish between patients with influenza and healthy subjects. Figure 2 shows an overview of an infectious screening system.

### 2.3. Sensor Fusion Combining RGB sensor and IRT for ROI Detection

A stable measurement of the body temperature and RR using an IRT needs a detailed ROI detection of facial landmarks (i.e., face, nose and mouth) because temperature is estimated at the facial area and respiration occurs at the nose and mouth. An RGB camera can detect facial landmarks finely using previous methods [20]. Therefore, we introduced a sensor fusion method to obtain facial landmarks in a thermal video determined by an RGB video.

**Figure 3:**
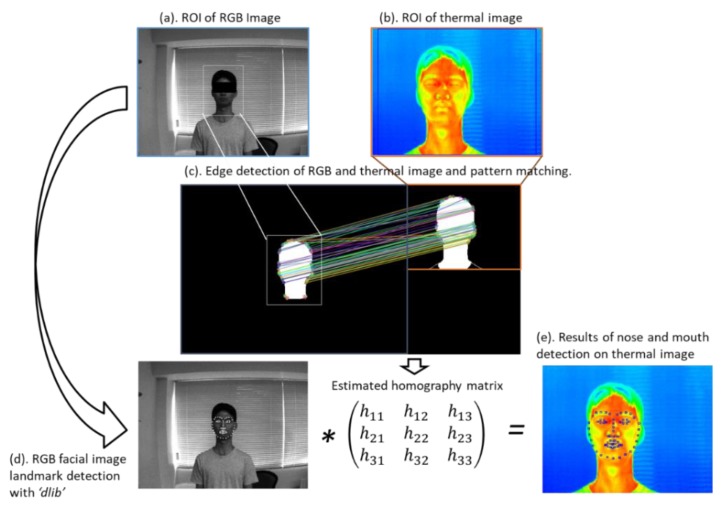
Feature matching for region-of-interest (ROI) detection in thermal image. The figure reproduced with copyright permission from Reference [14].

The facial landmarks in a thermal video are detected by homography of the RGB image coordinates of the nose and mouth, detected by “dlib” of an open-source library to thermal image coordinates. The homography between the images is represented by equation (1) and the homography matrix H is represented as

(1) $$H=\left(\begin{array}{ccc} h_{11} & h_{12} & h_{13}\\ h_{21} & h_{22} & h_{23}\\ h_{31} & h_{32} & h_{33} \end{array}\right),\;x_{thermo}=\frac{h_{11}x_{RGB}+h_{12}y_{RGB}+h_{13}}{h_{31}x_{RGB}+h_{32}y_{RGB}+h_{33}},\;y_{thermo}=\frac{h_{21}x_{RGB}+h_{22}y_{RGB}+h_{23}}{h_{31}x_{RGB}+h_{32}y_{RGB}+h_{33}},$$

where $x_{RGB}$, $y_{RGB}$, $x_{thermo}$ and $y_{thermo}$ are image coordinates in the RGB and thermal images. Each $h_{ij}\;\left(i,j=1,2,3\right)$ in Equation (1) is an element of the homography matrix H. Figure 3 shows a flowchart of image processing conducted to estimate the homography matrix H. Its standard is the face profile between the RGB and thermal images using pattern matching. First, from the RGB and thermal images shown in Figure 3a,b, the profile part is abstracted using the “grabcut” method [21] of OpenCV, to obtain the profile images shown in Figure 3c. The combination of coordinates between the images is found by obtaining the oriented fast and rotated BRIEF (ORB) characteristics of the two profile images and by performing a full search of the corresponding points from the characteristic points of each image obtained [22]. The homography matrix for the combination of image coordinates obtained is estimated using the random sample consensus method [23]. Finally, the facial landmarks in the thermal image (Figure 3e) are detected by applying the homography matrix to RGB’s facial landmarks (Figure 3d).

### 2.4. RGB Sensor Processing for HR Estimation Using Tapered Window, Signal Reconstruction based on Softsig and MUSIC Algorithm

**Figure 4:**
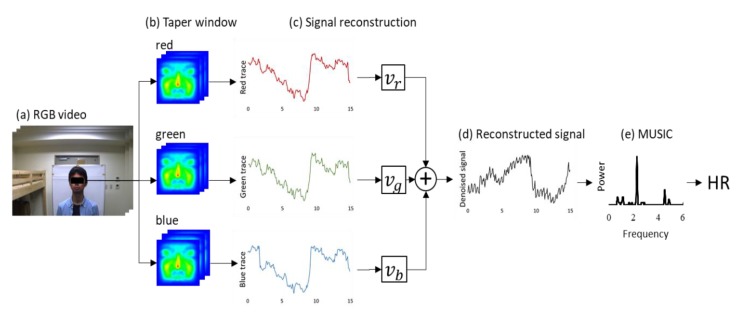
Block diagram of signal processing for HR estimation. (**a**) RGB video with ROI detected by OpenCV. (**b**) RGB ROI image applied to tapered window. (**c**) Raw RGB time-series data and reconstruction vector $V=\left(v_{r},v_{g},\;v_{b}\right)$ determined by kurtosis of spectra. (**d**) Reconstructed signal using *V*. (**e**) Power spectra obtained by MUSIC.

The fundamental method of HR estimation using an RGB camera has been described previously [15]. The RGB camera senses tiny color fluctuations in the facial skin with other noise. To remove the noise components, methods such as independent component analysis (ICA) and soft signature-based extraction (Softsig) [24] are used. In this study, we introduce the tapered window and signal reconstruction method into HR estimation for a stable measurement, which achieved an infection screening system. The observed RGB time-series data have components of heartbeat, motion artifact and noise from other light sources. The tapered window and signal reconstruction method is based on the Softsig demix heartbeat signal. Figure 4 shows an overview of HR estimation in this system.

Tapered window, which is a general window function, was applied to the detected facial ROI (Figure 4b). In facial ROI, the edge area suffers from the lag affected by the face tracker. On the other hand, the ROI center can achieve a stable tracking of the facial skin. Therefore, we adopted tapered window to weighted ROI to reduce the noise raised by facial tracking. A 1d-tapered window is represented as

(2) $$tapaer_{1d}\left(i\right)=\{\begin{array}{c} 0.5x\left(i\right)\left(1-\cos\left(\frac{2\pi i}{2m}\right)\right)\;\;\;\;\;\;\;\;\;\;\;\;\left(i=0,1,2,\ldots ,m-1\right)\\ 0.5x\left(i\right)\left(1-\cos\left(\frac{2\pi \left(n-i-1\right)}{2m}\right)\right)\;\;\;\;\;\;\;\left(i=n-m,\ldots ,n\right)\\ \;\;\;\;\;\;\;x\left(i\right)\;\;\;\;\;\;\;\;\;\;\;\;\;\;\;\;\;\;\;\;\;\;\;\;\;\left(otherwise\right), \end{array}$$

where *m* indicates the tapered portion and has a value of 0.05⋅n. To apply the tapered window to a 2d-image, the 2d-tapered window is expressed as

(3) $$tapaer_{2d}\left(x,\;y\right)=taper_{1d}\left(x\right)\cdot taper_{1d}\left(y\right),$$

where *x* and *y* are the x-coordinates and y-coordinates of ROI, respectively.

The aim of signal reconstruction is to find a reconstruction vector $V=\left(v_{r},v_{g},\;v_{b}\right)$ for extracting the heartbeat signal by utilizing the difference among RGB absorption. Reconstructing a BVP signal using three RGB channels to optimize a linear function for improving the signal-to-noise ratio. According to a previous study, the reflection strength of the heartbeat is referred to as the relation in G>B>R order among the RGB channels. Using this relation, signal reconstruction can be expressed as

(4) $$y\left(t\right)=v_{r}x_{r}\left(t\right)+v_{g}x_{g}\left(t\right)+v_{b}x_{b}\left(t\right),$$

where $v_{r},\;v_{g},\;\;\mathrm{and}\;v_{b}$ are the reconstruction vector. While this method is based on the Softsig method, we improved the determined method for vector V. To recover the pulse signal, we selected V to maximize the kurtosis of the spectra in the HR range of [0.75–4.0 Hz] (Figure 4c).

Finally, the MUSIC method was introduced to realize HR and RR measurements within a short time period. This method permits the realization of high-resolution HR and RR frequency estimation based on short-period measurement data Equation (5) expresses the spectrum estimation formula of the MUSIC method [14]:

(5) $$S_{MUSIC}\left(f\right)=\frac{1}{\sum_{k=M+1}^{p}{\left|e^{T}\left(f\right)W_{k}\right|}^{2}}\times \frac{1}{\delta f},$$

where $e\left(f_{i}\right)$ represents a complex sinusoidal wave vector and $W_{k}$ represents the eigenvector of the correlation matrix. This system applies the MUSIC method separately to the HR and RR time-series data obtained from the video. In the case of heartbeat, the peak of 0.75–3.0 Hz (45–180 beats per minute (bpm)) of the obtained spectrum was assumed to be the HR. 

### 2.5. IRT Sensor Processing for RR Estimation Using Nasal and Oral Breathing Decision based on SQI and MUSIC Algorithm and Body Temperature Estimation

**Figure 5:**
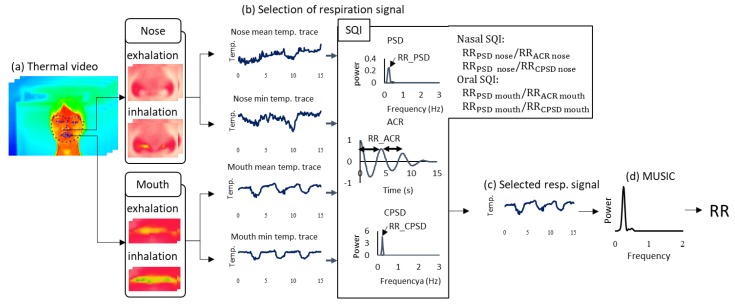
Block diagram of signal processing for respiration rate (RR) estimation. (**a**) Thermal video frame with facial landmark detected by the fusion sensor system described in Section 2. (**b**) Time-series data extracted from nasal and oral areas. (**c**) Respiration signal that chooses from four signals (b) based on SQI. (**d**) Power spectra obtained by MUSIC.

The current approach of respiration measurement using an IRT is based on nasal temperature change. However, mouth breathing is reported in 17% of the total population [25]. For a stable RR measurement using an IRT, we must also measure oral temperature changes and select nasal or oral temperature changes dependent on strongly including respiration. To choose nasal or oral breathing, we quantified temperature traces via nasal and oral areas using SQI. Moreover, the MUSIC algorithm achieved rapid measurement for RR estimation. Figure 5 shows an overview of the respiration measurement that introduces nasal and oral breathing measurement method and MUSIC algorithm.

First, the nasal and oral areas were detected using the fusion sensor system described in Section 2. The possible respiration signals were extracted by the two areas. The mean temperature fluctuation $x_{mean}\left(t\right)$ in each ROI and the min temperature fluctuation $x_{min}\left(t\right)$ in each ROI are expressed as

(6) $$x_{mean}\left(t\right)=\frac{1}{mn}\overset{m-1}{\underset{x=0}{\sum }}\overset{n-1}{\underset{y=0}{\sum }}I\left(x,y,t\right)\;x_{min}\left(t\right)=\underset{0<x<m-1,\;0<y<n-1}{\min}I\left(x.y,t\right),$$

where *I(x,y,t)* is the pixel temperature at the image coordinate (*x, y*) in the ROI and time *t, m* is the width of the ROI and n is the height of the ROI. $x_{mean}\left(t\right)$ and $x_{min}\left(t\right)$ include the respiration signals.

Second, the respiration signal is selected from nasal and oral temperature traces using the four extracted signals: $x_{mean\;nose}\left(t\right)$, $\;x_{min\;nose}\left(t\right)$, $\;x_{mean\;mouth}\left(t\right)$ and $x_{\min mouth}\left(t\right)$. Selection of the proposed respiration signal is conducted using the nasal SQI and oral SQI, based on the agreement of frequency estimated by power spectral density (PSD), autocorrelation (ACR) and cross-power spectral density (CPSD). The frequency of PSD using $x_{mean}\left(t\right)$ was estimated from the peak of power spectra from 0.1–0.75 Hz, to provide the range of RR measurement. The frequency of ACR using $x_{mean}\left(t\right)$ was estimated from the average peak interval. The frequency of CPSD using $x_{mean}\left(t\right)$ and $x_{min}\left(t\right)$ was estimated from the peak of cross-power spectra ranging from 0.1–0.75 Hz. If the temperature change in the nasal or oral area includes dominant respiration frequency, CPSD indicates the frequency by strengthening the respiration frequency between $x_{mean}\left(t\right)$ and $x_{min}\left(t\right)$ in the ROI. The following two rules are adopted sequentially:

1. Rule 1 (nasal SQI): If the ratio of $RR_{PSD\;nose}$ to $RR_{ACR\;nose}$ and that of $RR_{PSD\;nose}$ to $RR_{CSPD\;nose}$ obtained by the nasal area lie between 0.85 and 1.15, we select the nasal temperature change as the respiration signal. (This index shows that the nasal area includes the respiration signal because a ratio close to 1 indicates that the respiration frequency is dominant)
2. Rule 2 (oral SQI): If the ratio of $RR_{PSD\;mouth}$ to $RR_{ACR\;mouth}$ and that of $RR_{PSD\;mouth}$ to $RR_{CSPD\;mouth}$ obtained by the oral area lie between 0.85 and 1.15, we select the oral temperature change as the respiration signal. (This index shows that the oral area includes the respiration signal because a ratio close to 1 indicates that the respiration frequency is dominant)

If the two rules are not satisfied, we select nasal area as the respiration signal.

This system applies the MUSIC method separately to the HR and RR time-series data obtained from the video. In the case of respiration, the peak of 0.1– 0.75 Hz (6–45 bpm) of the spectrum obtained was assumed to be the RR. Temperature was also determined as the max facial temperature in the detected facial ROI using the sensor fusion technique.

### 2.6. SVM Discriminant Analysis to Predict Patients with Seasonal Influenza based on the Three Vital Signs Measured

Aiming at screening using features of HR, RR and body temperature of patients with infection, we proposed a classification model based on SVM. SVM is a method that predicts the separating hyperplane to maximize the margin between the two classes and achieves a high generalization capability. The SVM discriminant function is defined as

(7) $$\underset{w,\;\;w_{0},\;\xi }{\min}\left(\frac{1}{2}{\left\|w\right\|}^{2}+C\overset{N}{\underset{i=0}{\sum }}\xi_{i}\right)\;\mathrm{subject}\;\mathrm{to}\;\{\begin{array}{c} y_{i}f\left(x_{i}\right)\geq 1-\xi_{i}\\ \xi_{i}\geq 0 \end{array},$$

where w is a constant that indicates the SVM coefficients corresponding to HR, RR and temperature; $y_{i}$ is a category of health or infection; C is the penalty parameter and $\xi_{i}$ is the slack parameter; $f\left(x_{i}\right)$ is linear discriminant function formula $w\cdot x_{i}+w_{0}$. The calculation of SVM is performed using the MATLAB software.

### 2.7. Evaluation of the System in Laboratory and Clinical Settings

Laboratory and clinical testing of the system was conducted in 2019. Twenty-two healthy control subjects with no symptoms of fever (23.4 years of average age) participated in the laboratory test at the University of Electro-Communications. A total of 41 patients (45.0 years of average age) with symptoms such as influenza were included, who visited Takasaka Clinic, Fukushima, Japan. Their RR, HR and body temperature were measured using the contactless system; reference measurements were simultaneously obtained using a contact-type electrocardiogram (ECG) (LRR-03, GMS Co. Ltd., Tokyo, Japan) or pulse oximeter (SAT-2200 Oxypal mini, NIHONKOHDEN Co., Tokyo, Japan), clinical thermometer (TERUMO electric thermometer C230, TERUMO Co., Tokyo, Japan) and a respiration effort belt (DL-231, S&ME Inc.,Tokyo, Japan). It should be noted that, some patients may show increased heart rate due to white-coat hypertension. This study was approved by the Committee on Human Research of the Faculty of System Design, Tokyo Metropolitan University and the University of Electro-Communications. All subjects gave their informed written consent.

### 2.8. Statistical Analysis

The Bland–Altman plot and scatter plot were utilized for statistical and graphical proof of the agreement between the proposed method and reference method [26]. The reference vital signs were measured by ECG or a pulse oximeter for HR, respiration effort belt for RR and electronic thermometer for axillary temperature. The results from the SVM classification model were used to calculate the sensitivity, specificity negative predictive value (NPV) and positive predictive value (PPV). A leave-one-out cross-validation was performed to avoid overfitting.

## 3. Results

### 3.1. HR Measurements Using RGB Sensor in a Laboratory and Clinical Setting

**Figure 6:**
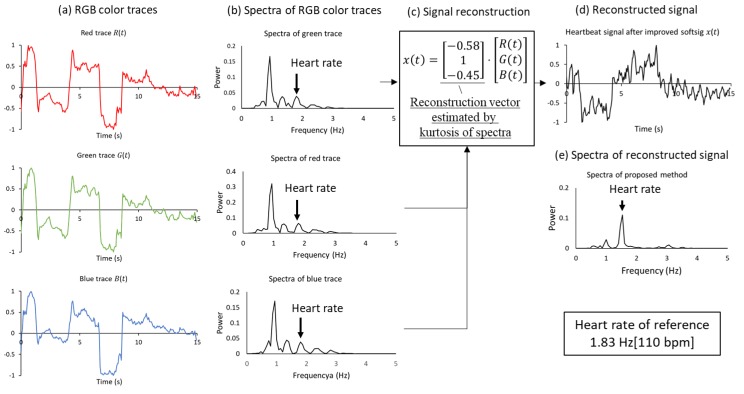
Recovery of heartbeat signal by applying tapered window and signal reconstruction. (**a**) RGB color traces obtained by RGB video. (**b**) Spectra estimated by Fast Fourier Transform (FFT). (**c**) Signal reconstruction determined through kurtosis of the spectra. (**d**), (**e**) Reconstructed signal and its spectra.

Figure 6 presents an example of signal recovery applied using the proposed method, by employing the tapered window and signal reconstruction based on Softsig. Raw traces of RGB color (Figure 6a) contained a dominant frequency of noise components, which can be observed by their spectra (Figure 6b), because the ground truth of HR measured by the pulse oximeter is 1.83 Hz. However, applying the proposed method, we can observe a clear peak of the HR frequency component in Figure 6e. This example shows the advantage of the proposed HR estimation.

**Figure 7:**
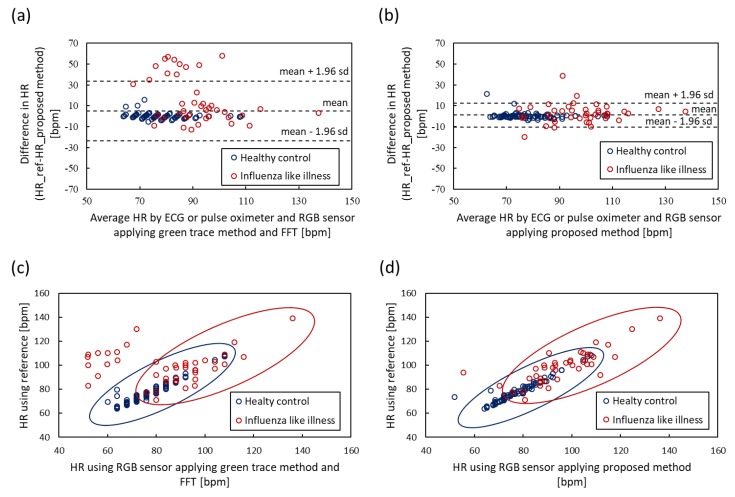
Bland–Altman plots and scatter plots of heart rate (HR) obtained by RGB sensor and electrocardiogram (ECG) or pulse oximeter. (**a**) Bland–Altman plot of raw green trace method applying FFT. (**b**) Bland–Altman plot of the proposed method applying tapered window, signal reconstruction and MUSIC. (**c**) Scatter plot of raw green trace. (**d**) Scatter plot of proposed method.

To evaluate the tapered window, signal reconstruction and MUSIC, we compared the proposed method to raw green trace, which uses only green channel and Fast Fourier Transform (FFT). The green trace method is a general method for estimating HR using an RGB camera. The ground truth of HR was measured by ECG and the pulse oximeter. We performed 15 s measurement four times against healthy control subjects and obtained 128 pairs of HRs from all subjects, which included 22 healthy control subjects and 41 patients with influenza-like symptoms. A comparison of HR estimation is shown in Figure 7. Figure 7a shows the Bland–Altman plot of green trace applying FFT. The 95% limits of agreement ranged from -23.5 to 33.4 bpm (standard deviation σ=14.5) and the root mean square error (RMSE) was 15.3. Figure 7c shows the scatter plot of the green trace method; the Pearson correlation coefficient was 0.48. Figure 7b shows the Bland–Altman plot of the proposed method, which applies the tapered window, signal reconstruction and MUSIC. The 95% limits of agreement ranged from -10.4 to 12.6 bpm (standard deviation σ=5.85) and RMSE was 5.93. Figure 7d shows the scatter plot of the proposed method; the Pearson correlation coefficient was 0.87. The results showed that the proposed method can reduce the 95% limits of agreement from [−23.5, 33.4] to [−10.4, 12.6] bpm. Especially, the result of patients with influenza-like illness (red circle) was improved because the experiment at a clinic is close to a real-world setting.

### 3.2. RR and Body Temperature Measurements Using IRT at a Laboratory and Clinical Settings

**Figure 8:**
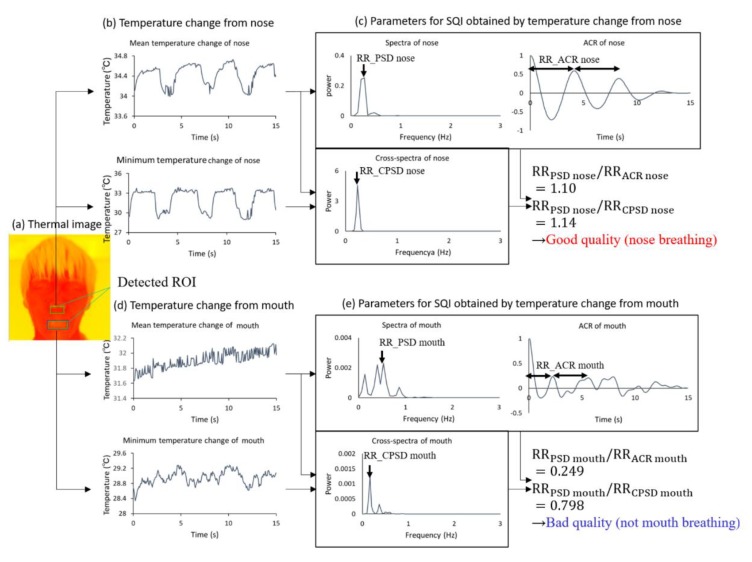
Determination of respiration signal applying nasal and oral breathing decision based on SQI. (**a**) Thermal facial image with ROI. (**b**) Mean and minimum temperature fluctuations in nasal area. (**c**) SQI parameter obtained by power spectral density (PSD), autocorrelation (ACR) and cross-power spectral density (CPSD) of nasal temperature changes. (**d**) Mean and minimum temperature fluctuations in oral area. (**e**) SQI parameter obtained by PSD, ACR and CPSD.

Figure 8 shows an example of the signal selection applied by the proposed method, which is detailed in Section 2. The mean and minimum temperature changes in each ROI are shown in Figure 8b,d. To determine the respiration signal from four signals, we calculated the SQI parameters, which included the PSD, ACR and CPSD of each signal (Figure 8c,e). Using the SQI parameters, we chose the respiration signal.

**Figure 9:**
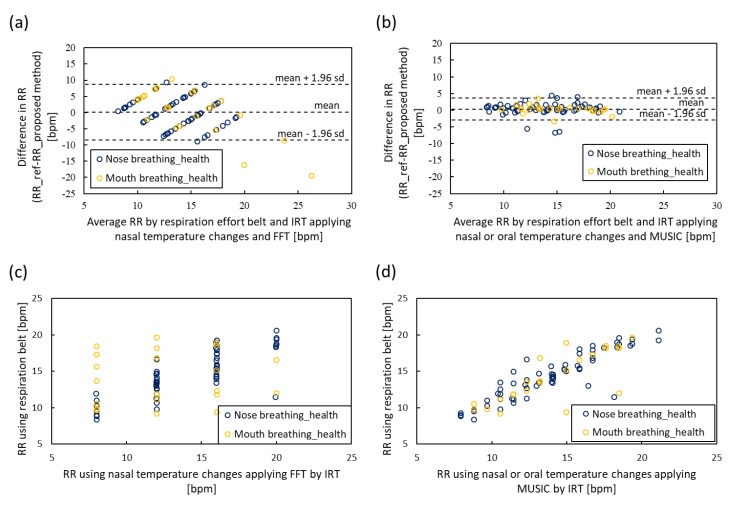
Bland–Altman plots and scatter plots of RR obtained by infrared thermography (IRT) sensor and respiratory effort belt. (**a**) Bland–Altman plot of nasal temperature change under the application of FFT. (**b**) Bland–Altman plot of the proposed method applying nasal or oral signal selection using SQI and MUSIC. (**c**) Scatter plot of nasal temperature change under FFT application. (**d**) Scatter plot of the proposed method.

To evaluate the nasal or oral breathing decision based on SQI and MUSIC, we compared the proposed method with the raw temperature change in the nasal area applied to FFT, which is a general method for estimating RR using IRT. The ground truth of RR was measured using the respiratory effort belt. We performed 15 s measurement four times and obtained 88 pairs of RRs from 22 healthy control subjects, including 6 subjects with nose clip for instructing subjects to mouth breathing. A comparison of RR estimation is shown in Figure 9. Figure 9a shows the Bland–Altman plot of nasal temperature change. The 95% limits of agreement ranged from -7.60 to 7.99 bpm (standard deviation σ=3.98) and the RMSE was 3.98. Figure 9c shows the scatter plot of nasal temperature change; the Pearson correlation coefficient was 0.53. Figure 9b shows the Bland–Altman plot of the proposed method. The 95% limits of agreement ranged from -2.97 to 3.67 bpm (standard deviation σ=1.68) and the RMSE was 1.73. Figure 9d shows the scatter plot of the proposed method; the Pearson correlation coefficient was 0.87. The results showed that the proposed method can reduce the 95% limits of agreement from [−7.60, 7.99] bpm to [−2.97, 3.67] bpm.

**Figure 10:**
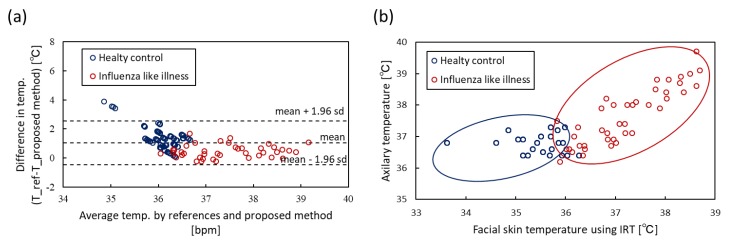
Bland–Altman plots and scatter plots of body temperature obtained by IRT sensor and electric thermometer. (**a**) Bland–Altman plot. (**b**) Scatter plot.

Facial temperature, which is estimated by ROI detection using sensor fusion, was also evaluated. The ground truth of the temperature was measured using an electric thermometer. From all subjects, which included 22 healthy control subjects and 41 patients with influenza-like symptoms, a comparison of temperature estimation is shown in Figure 10. Figure 10a shows the Bland–Altman plot of temperature. The 95% limits of agreement ranged from -0.45 to 2.56 ºC (standard deviation σ=0.77) and the RMSE was 1.30. Figure 10b shows the scatter plot; the Pearson correlation coefficient was 0.71.

### 3.3. Classification of Healthy Control Subjects and Influenza Patients

**Figure 11:**
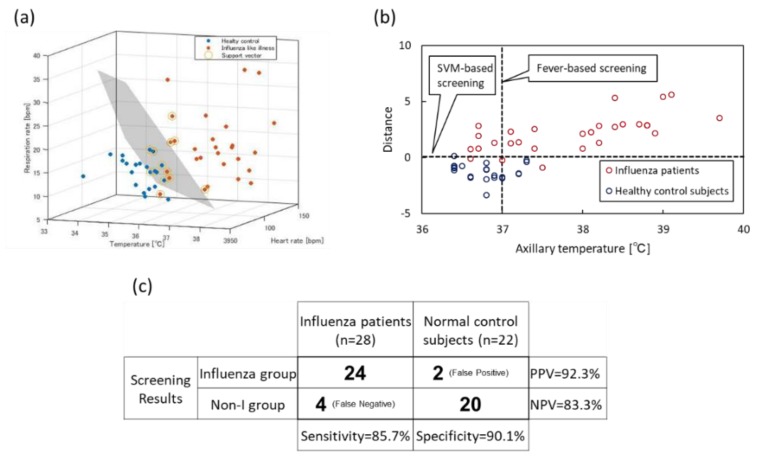
Classification model based on Support Vector Machine (SVM). (**a**) SVM classification. (**b**) Confusion matrix.

SVM established a classification model using three vital signs, including HR, RR and temperature, estimated by RGB and IRT sensors. The vital signs were measured for 22 healthy control subjects and 28 influenza patients (45.5 years of average age) diagnosed as influenza using virus isolation from all 41 patients with influenza-like symptoms. Figure 11a illustrates the distribution of the vital signs (22 blue dots: healthy control subjects, 28 red dots: influenza patients) and the separating hyperplane obtained by SVM using all data. SVM classification using the three vital signs achieved more accurate screening than fever-based classification (Figure 11b). Figure 11c presents the result obtained through leave-one-out cross-validation. The sensitivity, specificity, NPV and PPV were 85.7%, 90.1%, 83.3% and 92.3%, respectively. The fever-based screening using an electric thermometer was adopted to compare SVM classification. The sensitivity and specificity were 60.7% and 86.4%, respectively.

## 4. Discussion and Conclusions

The outbreak of 2019-nCoV was first reported in Wuhan, China, in December 2019 and was confirmed to have spread to more than 110 countries as of March 2020. When such a novel virus outbreaks, enhanced public health quarantine and isolation is essential. For this purpose, we developed a multiple vital sign measurement system for the mass screening of infected individuals in places of mass gathering. In this study, we focused on developing our system to measure three vital signs, to achieve automation, stability and swiftness for practical use in real-world settings. From a technical perspective, we proposed specific signal and image processing methods for highly reliable vital sign measurements and compared them with conventional methods (Table 1 and Table 2). Tapered window, RGB signal reconstruction and MUSIC were applied for HR measurement. Automatic ROI tracking using sensor fusion and nasal or oral breathing selection using SQI and MUSIC were applied for HR measurement. The proposed method showed agreement with their reference devices (HR: [−10.4, 12.6] bpm, RR: [−2.97, 3.67] bpm, temperature: [−0.449, 2.56] °C). The reliability and stability of our system on vital sign measurement were significantly improved. 

**Figure 12:**
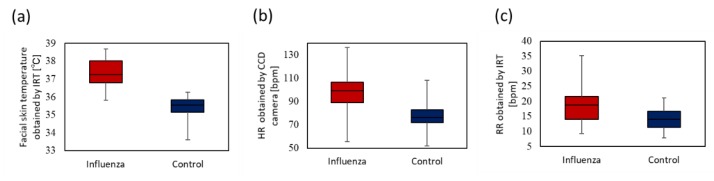
Box plot of vital signs between influenza patients and healthy control subjects. (**a**) Facial skin temperature. (**b**) HR. (**c**) RR.

Moreover, we tested multiple vital sign-based screening in a laboratory and a clinic. The proposed method’s sensitivity and specificity (85.7%, 90.1%) were found to be higher than those of fever-based screening (60.7%, 86.4%). The tendency of the three vital signs measured by healthy control subjects and influenza patients is shown in Figure 12. The medians of facial skin temperature of influenza patients and healthy control subjects were 37.3 and 35.5 °C, respectively. The medians of HR of influenza patients and healthy control subjects were 99.3 and 76.4 bpm. The medians of RR of influenza patients and healthy control subjects were 18.9 and 14.0 bpm. Each vital sign of patients with influenza was found to be elevated. This contributed to improvement in SVM classification based on the three vital signs.

However, the proposed method has some limitations. The ROI detection of sensor fusion may fail when the background has the color of skin or hair. In terms of the classification test based on SVM, the facial skin temperature may include the influence of the ambient environment. The measurement environment at a laboratory is different from that at a clinic, even at the same ambient temperature. This causes a difference in facial skin temperature regardless of the seasonal influenza. Therefore, we need to develop environment-invariant temperature estimation using an IRT.

In conclusion, we proposed automatic, stable and rapid HR, RR and body temperature measurements using an RGB-thermal sensor and its application for the screening of infectious diseases. This method introduces (1) the sensor fusion approach for the detection of detailed facial landmarks in a thermal image, (2) HR estimation, which introduces tapered window, signal reconstruction and MUSIC and (3) RR estimation, which implements nasal or oral breathing selection using SQI and MUSIC. Moreover, we demonstrated a classification model based on SVM using healthy control subjects and patients with seasonal influenza. The results indicate that the proposed method is indispensable for the high performance of contactless multiple vital sign measurements for infection screening.

## Figures and Tables

**Table 1:** Comparison of proposed RGB signal reconstruction method with conventional green trace method on HR measurement.

HR	RMSE	Bland–Altman	Pearson Correlation
RGB signal reconstruction and MUSIC	5.93	The 95% limits of agreement -10.4 to 12.6 bpm (σ=5.85)	0.87
Green trace alone and FFT	15.30	The 95% limits of agreement -23.5 to 33.4 bpm (σ=14.5)	0.48

**Table 2:** Comparison of proposed Nasal/oral SQI method with conventional nasal alone method on RR measurement.

RR	RMSE	Bland–Altman	Pearson Correlation
Nasal or oral SQI and MUSIC	1.73	The 95% limits of agreement -2.97 to 3.67 bpm (σ=1.68)	0.89
Nasal and FFT	3.98	The 95% limits of agreement -7.60 to 7.99 bpm (σ=3.98)	0.53
